# Multi-Hop Routing Mechanism for Reliable Sensor Computing

**DOI:** 10.3390/s91210117

**Published:** 2009-12-11

**Authors:** Jiann-Liang Chen, Yi-Wei Ma, Chia-Ping Lai, Chia-Cheng Hu, Yueh-Min Huang

**Affiliations:** 1 Department of Electrical Engineering, National Taiwan University of Science and Technology, Taipei, Taiwan; E-Mail: lchen@mail.ntust.edu.tw; 2 Department of Engineering Science, National Cheng Kung University, Tainan, Taiwan; E-Mail: n9897106@mail.ncku.edu.tw; 3 Department of Computer Science & Information Engineering, National Dong Hwa University, Hualien, Taiwan; E-Mail: m9321502@ems.ndhu.edu.tw; 4 Department of Information Management, Naval Academy, Kaohsiung, Taiwan

**Keywords:** reliability, sensor computing, cluster mechanism, routing algorithm, service lifetime

## Abstract

Current research on routing in wireless sensor computing concentrates on increasing the service lifetime, enabling scalability for large number of sensors and supporting fault tolerance for battery exhaustion and broken nodes. A sensor node is naturally exposed to various sources of unreliable communication channels and node failures. Sensor nodes have many failure modes, and each failure degrades the network performance. This work develops a novel mechanism, called Reliable Routing Mechanism (RRM), based on a hybrid cluster-based routing protocol to specify the best reliable routing path for sensor computing. Table-driven intra-cluster routing and on-demand inter-cluster routing are combined by changing the relationship between clusters for sensor computing. Applying a reliable routing mechanism in sensor computing can improve routing reliability, maintain low packet loss, minimize management overhead and save energy consumption. Simulation results indicate that the reliability of the proposed RRM mechanism is around 25% higher than that of the Dynamic Source Routing (DSR) and *ad hoc* On-demand Distance Vector routing (AODV) mechanisms.

## 1. Introduction

Recent advances in MEMS (Micro-Electro-Mechanical Systems) technology and wireless communications have led to small and low-cost sensors with increasingly powerful processing and networking capabilities. Sensor networks may comprise many sensor types, capable of monitoring a diversity of surrounding conditions, including temperature, humidity, lightning condition, pressure, noise levels, the presence or absence of particular objects and the object properties such as speed, direction and size. Additionally, many various domain applications, such as factory automation, chemical pollution monitoring, healthcare, and security adopt sensor computing [1–4].

**Figure 1.**
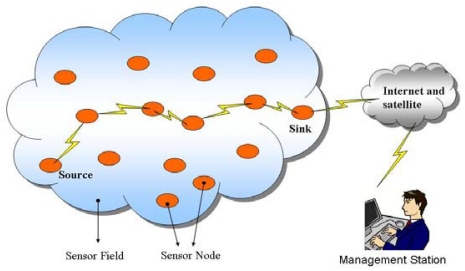
Communication architecture of sensor computing.

Figure 1 illustrates the communication architecture of wireless sensor computing. Up to thousands of sensor nodes are spread across a geographical area to monitor ambient conditions as mentioned. They cooperate with each other to form a sensing network, providing access to surrounding information anytime, anywhere. A sink may function as a powerful stationary sensor node, or a mobile hardware device carried by users to gather all sensing messages sent from multiple sensor nodes. While gathering messages successfully, sinks process and forward essential data to administrators *via* communication channels.

Sensor computing is limited by extremely constrained resources, such as storage, computation capability, radio model and energy. These limitations affect the types of routing mechanisms that can be efficiently deployed. Sensor nodes are generally powered by batteries, and these are often very difficult to change or recharge in inaccessible terrains. The power consumption in wireless sensor computing can be categorized into two parts, *i.e.*, communication and computation. Among these, communication consumes the most power. Hence, reducing the number of unnecessary transmissions is the best way to save energy consumption and prolong the lifetime of the sensor service network [5].

**Figure 2.**
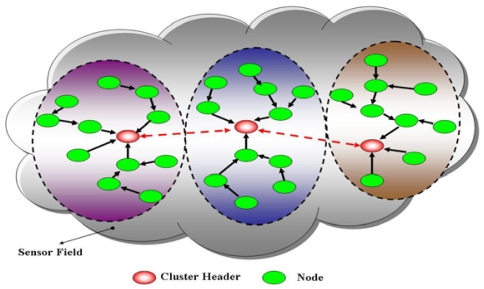
Cluster-based architecture.

Many various routing protocols, such as *ad hoc* On-demand Distance Vector (AODV), Dynamic Source Routing (DSR), have been proposed for *ad hoc* networks [6,7]. The performance of these approaches has been analyzed and compared with each other. Routing protocols for *ad hoc* networks are generally classified into three parts, namely on-demand, table-driven and hybrid. The route in the on-demand routing protocol is identified only when the source node is needed to send packets, and no destination address is given. Although utilizing less bandwidth to discover the routing path and minimize the overhead of the network, on-demand mechanisms have a higher end-to-end average delay. Oppositely, table-driven routing protocols discover routing paths and maintain routing tables occasionally even if the network is not in use. Although the latency of discovering the routing path is low in table-driven routing protocols, the amount of control packets along with new or broken nodes generates a high network overhead. Additionally, table-driven routing protocols expend more bandwidth usage for maintaining routing tables. The hybrid mechanism is a cluster-based routing protocol that exploits both the other two protocols. Figure 2 illustrates cluster-based routing protocols dividing all nodes into many clusters, applying a proactive protocol within clusters and a reactive protocol between clusters. A cluster-based structure not only restricts the message flooding scope, but also elects a cluster header in every cluster to exchange routing information. The structure reduces the overhead of the network and bandwidth usage, thus saving energy, and is appropriate for a wireless sensor computing.

Current research on routing in sensor computing focuses on maximizing the service lifetime, enabling scalability for large number of sensors and supporting fault tolerance for battery exhaustion and broken nodes [8]. A wireless network of sensor nodes is inherently exposed to various sources of unreliable communication channels and node failures. Sensor nodes have many failure modes [9]. Each failure degrades the network performance. This study proposes a novel mechanism involving a hybrid cluster-based routing protocol for sensor computing that selects the most reliable routing path. The proposed mechanism can improve routing reliability, maintain low packet loss, minimize management overhead and save energy consumption.

The rest of this paper is organized as follows. Section 2 provides the background knowledge of wireless sensor computing related work on reliability and cluster-based routing in sensor networking. Section 3 presents the proposed mechanism and algorithm. Section 4 then describes the simulation and implementation, and analysis of the results. Conclusions are finally drawn in Section 5.

## 2. Background Knowledge

Several related issues should be introduced in the design and construction of the proposed mechanism. In particular, the background knowledge about wireless sensor networks and reliability are very significant.

### 2.1. Wireless Sensor Network (WSN)

A sensor network comprises many sensor nodes, which are randomly deployed in inaccessible areas around a phenomenon without predetermination. A sensor node consists of four basic components namely sensing unit, processing unit, transceiver unit and power unit. The sensing units usually comprise two subunits, namely sensors and analog-to-digital converters (ADCs). The analog signals produced by the sensors are converted into digital signals by the ADC, and then fed into the processing unit. The processing unit, which is generally linked with a small storage unit including ROM and RAM, manages the procedures to execute the assigned jobs. A transceiver unit connects the node to the network, and communicates with other nodes. One of the most important components of a sensor node is the power unit. Power units may be only supported by batteries, or by solar cells that act like a power generator without recharging. Moreover, some other application-dependent components may be attached. A mobilize is needed when the sensor nodes need to move to carry out the assigned jobs. Advances in hardware technology mean that nodes, including all subunits are now smaller matchboxes device. Some additional stringent constraints for sensor nodes are low power consumption, operation in high dense sensor network, low production cost and adaptation to the environment.

### 2.2. WSN Routing Mechanism

Routing is a key issue in sensor computing. A pair of nodes not within the transmission range can communicate with each other by other intermediate nodes used as relay nodes. The selection of intermediate nodes to send a message is called routing. In other words, the routing process is to construct a path between the source node and destination node that is not within the transmission range. Many routing researches have been proposed for sensor networks. A routing protocol for sensor networks should have scalability, data aggregation, network dynamics, low complexity, energy-efficiency, fault tolerance and multiple paths [10].

**Figure 3.**
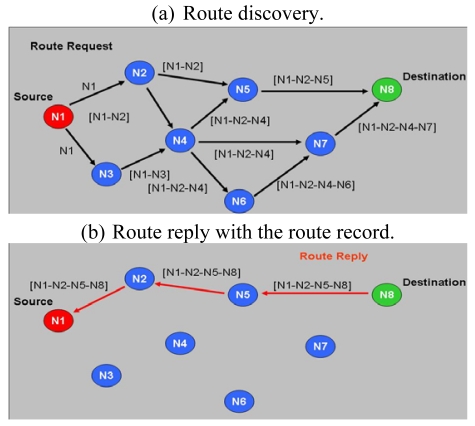
DSR routing operations.

**Figure 4.**
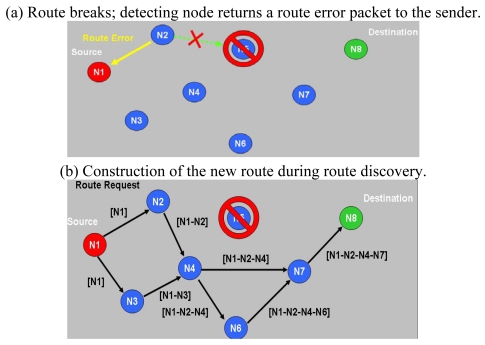

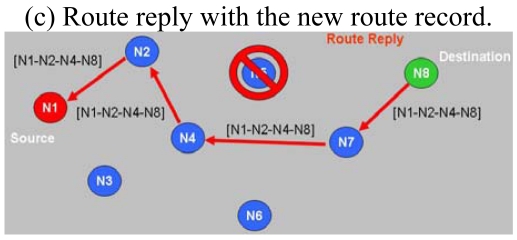
Route maintenance in DSR protocol.

In on-demand routing mechanisms, the route is discovered only when needed by the source node, minimizing the network overhead at the cost of a slow response. One such mechanism is DSR (Dynamic Source Routing), which is an on-demand routing protocol. DSR allows a network to be completely self-organizing and self-configuring, without the need for any existing network infrastructure or administration. The protocol consists of the two main mechanisms, namely “Route Discovery” and “Route Maintenance”, which work together to allow nodes to discover and maintain routes to arbitrary destinations in the *ad hoc* network. All aspects of the protocol operate entirely on-demand, allowing the routing packet overhead of DSR to scale automatically to only that needed to react to changes in the routes currently in operation. Determining the source routes requires obtaining the address of each device between the source and destination during route discovery. The source demands a routing path by flooding request packets in the networks. The accumulated path information is cached by nodes processing the route discovery packets. A node denotes a route with complete information of the destination node it has traversed, as shown in Figure 3. This may result in a high overhead for long paths. Figure 4 shows a scenario in which a route breaks; the detecting node returns a Route Error packet to the original sender. The sender can invoke Route Discovery again to obtain a new route.

Clustering means that grouping all wireless sensor nodes into many clusters [11,12]. Partitioning the whole sensor network into small regions, can turn a network into an easily controllable and manageable infrastructure. Clustering provides a good framework for power control, low interference and efficient channel utilization. Clusters are generally formed in two stages: (1) a cluster header is selected at random or by a pre-defined method; (2) cluster headers and the member nodes interact to form a group named cluster. A cluster performs information filtering, data fusion and aggregation such as periodic calculation of the average temperature of its coverage area, and effectively reduces communication overhead. The amount of control traffics is limited within each cluster, helping reduce the energy consumption. Since the cluster header must manage all nodes belonging to it, excessive operations of header may quickly cause energy exhaustion. Hence, the nodes within a cluster take turns based on a round-robin strategy to act as the cluster header. The strategy can also be determined by a node's connectivity relationships or power level, in order to balance the energy consumption and extend the system lifetime.

### 2.3. Network Reliability

**Figure 5.**
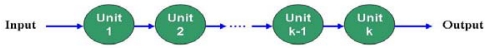
Network elements linked with serial configuration.

The network design problem is to arrange these components such that a given set of traffic requirements are met at the lowest cost. This problem is known as network optimization, and concerns throughput, delay and reliability. Reliability is the probability that a network will perform satisfactorily for a given period of time when adopted under specified operating condition. The topological connectivity generally determines the network reliability. This study focuses on individual elements. Figure 5 shows the series-connected units.

If the units do not interact, then the failures are independent, and the system reliability *R_s_*(*t*) denotes the product of the reliabilities of the individual constituent units. The function is set as follows:

$$R_{s}(t)=\prod_{i=1}^{k}R_{i}(t)=\prod_{i=1}^{k}\left[1-F_{i}(t)\right]$$

In this function, *R_i_*(*t*) and *F_i_*(*t*) are the reliability and failure distribution functions, respectively, of the *i*th system unit.

**Figure 6.**
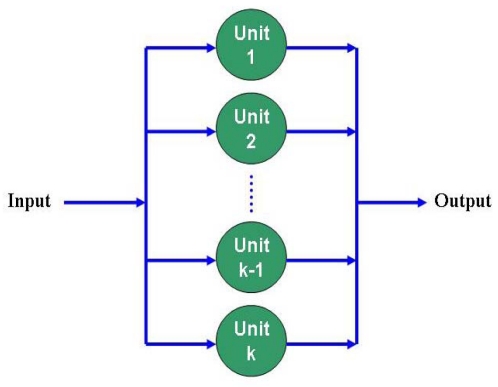
Network elements connected with parallel configuration.

In a parallel network, a number of similar individual components are connected in parallel. Figure 6 shows a parallel network with *k* units. To derive the reliability function *R_p_*(*t*) for the parallel network, the following two assumptions are made: (1) all units are active and share the network load, and (2) all components are statistically independent. For no identical components, the failure distribution function *F_p_*(*t*) at time *t* is given by:

$$F_{p}(t)=\prod_{i=1}^{k}F_{i}(t)$$

where *F_i_*(*t*) = 1-*R_i_*(*t*) denotes the failure distribution of the *i*th component.

Since *R_p_*(*t*) + *F_p_*(*t*) =1, the parallel-configuration reliability is given by

$$R_{p}(t)=1-F_{p}(t)=1-\prod_{i=1}^{k}\left[1-R_{i}(t)\right].$$

The sensor network has highly constrained resources such as storage, computation capability, radio model and energy, and these limitations affect the routing mechanisms that can be efficiently deployed. Sensor nodes have many failure modes. Each failure degrades the network performance. An excellent and complete routing protocol and algorithm for handling reliability routing path of wireless sensor computing can be obtained by combining the advantages and disadvantages of the systems described in the above related works. Therefore, this study proposes a novel mechanism that adopts a hybrid cluster-based routing protocol for sensor computing to select the best reliable routing path.

## 3. Proposed Reliable Routing Mechanism

This section introduces several basic assumptions for the proposed network model. The cluster-based border gateway routing protocol divides the routing into two schemes, intra-cluster routing and inter-cluster routing. The cluster structure describes in detail the handling of the reliability routing paths of wireless computing.

This study is based on the following assumptions:

- All sensor nodes are stationary at their initial locations after they are deployed.
- Every sensor node has limited energy.
- The sensor network is organized into clusters with any clustering method, and every node has a unique identity for determining its cluster.

A large-scale region is covered by a large number of homogeneous sensor nodes. These sensor nodes communicate with each other through short-range radios, and wireless channels are bidirectional. Long distance data forwarding is achieved across multiple hops.

### 3.1. Reliable Intra-cluster Routing

**Figure 7.**
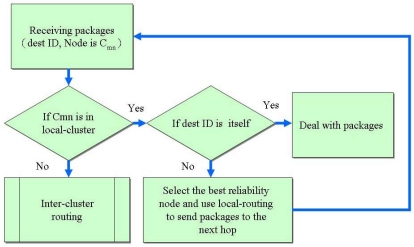
Intra-cluster routing algorithm.

The intra-cluster routing is based on slightly modified table-driven routing mechanisms like Destination Sequence Distance Vector (DSDV). In this routing mechanism, the node periodically exchange routing information. A node broadcasts its table to its neighbors once its routing table changes. This approach limits the exchange range within the cluster and its next hop to reduce the control overhead and interference with the shared media. Every node maintains routing information of other nodes within its cluster, including destination, next hop, cluster id of destination node, metric, sequence number and accumulating the reliability of each node between the source and destination during route discovery. Moreover, the border node in each adjacent cluster is added to the local routing table, enabling routing to adjacent clusters. The above routing information is adopted for routing selection in intra-cluster routing. No cluster head is elected in charge of transmission and routing maintain, thus avoiding the bottlenecks and reducing the control packets of choosing the cluster head. Figure 7 illustrates the intra-cluster routing algorithm.

**Figure 8.**
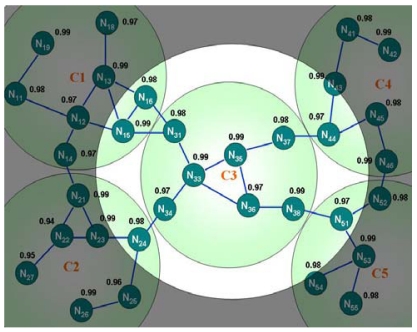
Intra-cluster routing.

Table 1 shows the routing information of node N_33_ given in Figure 8. Node N_33_ can easily deliver the packets to node N_38_ by the routing information in Table 1. When the packets are transmitted to N_13_, which is located on the adjacent cluster C1, node N_33_ has no route to N_13_ in its routing table.

However, node N_33_ knows that destination node N_13_ belongs to cluster C1, and discovers that the route to C1 is available in the second entry of Table 1 (selected by the best reliable path). Accordingly, node N_33_ sends the packets to the next hop N_31_. The packets are sent to node N_15_ followed by node N_13_ when this step is applied iteratively. The Intra-cluster routing of Cluster C1 is similarly adopted to convey the packets to N_19_ iteratively.

**Figure 9.**
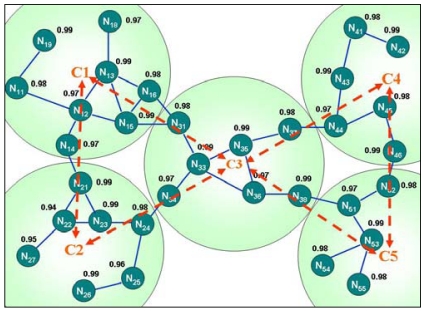
Relationship between local cluster and adjacent clusters.

Moreover, these gateways, which are the border nodes to other clusters, are selected while the intra-cluster routing scheme is being built. Hence, no extra effort is required for gateway selection, and overhead is reduced. Figure 9 shows an example of this scheme.

Cluster C3 can communicate with C1 *via* gateway N_15_, and with C4 *via* N_44_. Moreover, two adjacent clusters may have more than one border node, which constitute multi-path gateways to adjacent clusters. Multi-path gateways enhance the fault tolerance, since the sensor node failure caused by blockage due to power depletion, physical damage or the power-saving schedule would not affect the inter-cluster communication in the sensor network. Additionally, multi-path gateways enhance the load balance, avoiding wear problems. Figure 9 shows that cluster C3 can communicate with C1 by passing either gateway N_15_ or N_16_. The proposed algorithm selects the best reliable node by default. The multi-path routing enhances the robustness of the wireless sensor network. If one gateway fails, then the packets can be sent *via* another gateway.

### 3.2. Reliable Inter-Cluster Routing

**Figure 10.**
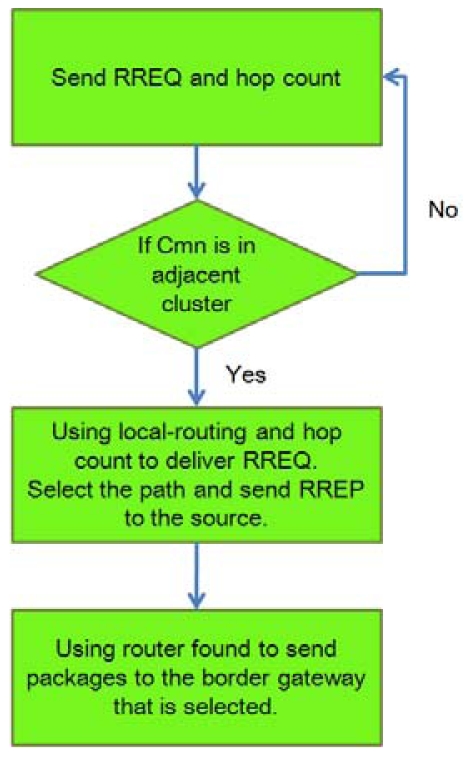
Inter-cluster search for routing algorithm.

**Figure 11.**
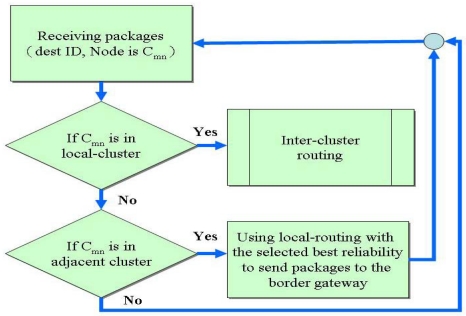
Inter-cluster routing algorithms.

Inter-cluster routing is achieved by extracting the relationships between clusters from the local routing table, which includes the next hop nodes in clusters adjacent to the cluster edge. The inter-cluster routing is obtained by exchanging the relationships between clusters. Discovering inter-cluster routes when the route is demanded can reduce the overhead of constructing and maintaining Inter-cluster routing. On-demand routing protocols such as DSR adopt flooding as route discovery method; no previous routes are available to guide the packets to the destination. Nevertheless, these methods increase not only the route discovery latency but also the overhead, depending on the flooding range. In this study, the cluster-based border gateway routing protocol is slightly modified to overcome this problem and provide the reliability information of the routing path. When the demand for an inter-cluster route occurs, the source node sends the inter-cluster Route REQuest packet (RREQ packet) in unicast mode to the border nodes to obtain the adjacent cluster's intra-cluster routing information, from which the inter-cluster route can be created. Figures 10 and 11 show the routing algorithm.

**Figure 12.**
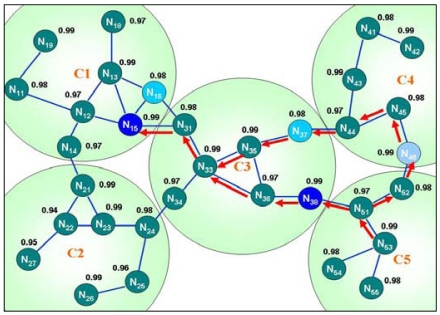
Inter-cluster routing.

For instance (as shown in Figure 12), node N_53_ in C5 wishes to communicate with node N_12_ in C1, but the route to N_12_ or C1 cannot be found in N_53_'s intra-cluster routing table. The inter-cluster RREQ packet is sent to the border nodes N_46_ and N_38_ to obtain the adjacent cluster's intra-cluster routing information. The inter-cluster RREQ is cached in the node that received it, and is not removed until the expiry time is due.

The RREQ packet is dropped when the node receives the inter-cluster RREQ with the same source and sequence number. If the route cannot be found in the border node's intra-cluster routing table, then the border node sends the inter-cluster RREQ again to other border nodes. This procedure is repeated as needed until the edge of the sensor network is reached. In this case, the path to C1 can be identified from the intra-cluster routing table of the border nodes in C3, and the border node sends an inter-cluster route reply RREP packet back to the source node; the route to C1 is added into the intra-cluster routing table of the nodes located on the reverse path of the inter-cluster RREP traveled. That is, C5 obtains the path to C1 by passing C3 or C4, but C3 has the best reliable path; moreover, if the N_38_ nodes failure or other unavoidable reasons, then the route from N_53_ to N_12_ is reconstructed along the path through N_46_, N_37_ and N_15_.

### 3.3. Routing Strategic Decision

**Figure 13.**
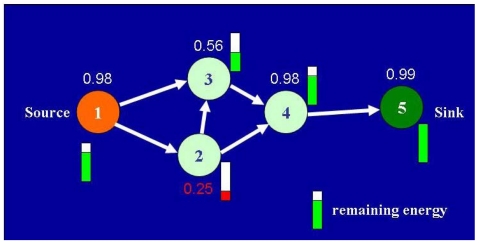
Energy consumption decrease the reliability.

The nodes exchange routing information in the intra-cluster routing mechanism. A node broadcasts its table to its neighbors when its routing table changes. Each node maintains routing information of other nodes within its cluster, including destination, next hop, cluster id of destination node, metric, sequence number, and obtains the reliability of each node between the source and destination during route discovery. Moreover, the border node in each adjacent cluster is added to the local routing table, enabling routing to adjacent clusters. The routing information mentioned above is adopted for routing selection in intra-cluster routing. Each routing table of node focuses on the reliability and hop count. The reliability value is integrated with energy consumption and various surrounding conditions such as temperature, humidity, pressure and noise levels. Figure 13 shows an example of low-energy nodes decreasing the reliability.

**Figure 14.**
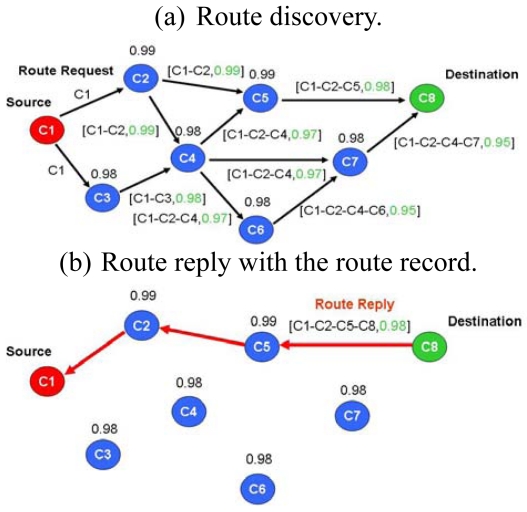
DSR operations.

In the inter-cluster routing mechanism, the routing path, value of reliability and hop count appended to the RREQ packet. This routing information is adopted for routing selection in inter-cluster routing (as shown in Figure 14). The hop count can be adopted to minimize the length of the routing path while maintaining reasonable reliability.

## 4. Performance Analysis and Discussion

This section first introduces the simulation network model. The simulation metrics for measuring the performance of the proposed Reliable Routing Mechanism (RRM) are them presented. The performance comparisons among the proposed RRM, AODV [6] and DSR [7] mechanisms are then presented.

### 4.1. Simulation Environment

The simulation environment has 200 sensor nodes deployed in a 2,000 m × 2,000 m area. A two-ray ground is adopted as the radio propagation model and an omni-directional antenna with unity gain. Each data packet size has 64 bytes, query packet and the others are 36 bytes. The source node generates one data packet per second. Each simulation time lasts for 200 seconds. The power consumptions of the nodes for transmitting, receiving and idling are approximately 660 mW, 359 mW and 35 mW respectively.

The performance of the RRM mechanism was measured and compared with two existing well-known protocols, AODV and DSR, using the packet delivery ratio, reliability of delivery, control overhead, average latency, throughput and system lifetime.

- Packet delivery ratio refers to the ratio of the number of data packets received by the destination to the number sent by the source.
- Reliability of delivery refers to the reliability of path between the destination and the source.
- Routing overhead refers to the total number of packets generated during discovering routing paths.
- Average latency refers to the average time between the time when a source transmits a packet and the time when a sink receives the packet.
- Throughput refers to the number of bytes passing through a data communication system per period of time.
- System lifetime refers to the time that the network is alive.

### 4.2. Packet Delivery Ratio

**Figure 15.**
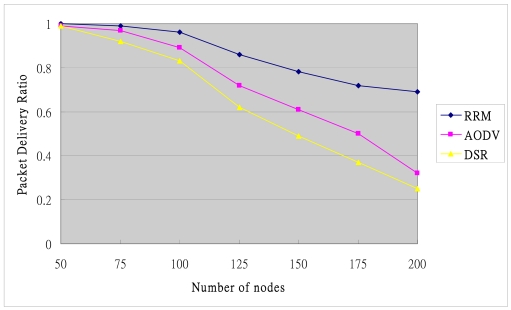
Packet delivery ratio.

The Packet Delivery Ratio falls with the rise in the number of sensor nodes in a wireless sensor computing, because the high congestion of routing overhead around sensor nodes causes packets to be dropped. Figure 15 plots the Packet Delivery Ratio of DSR and AODV, showing a big fall when the number of nodes in the network rises above 100. This big descent occurs because DSR and AODV broadcast RREQ throughout the network to discover a new route. In the proposed RRM mechanism, if one node of a routing path fails, then the source or border node immediately selects a cached routing path, thus improving the Packet Delivery Ratio.

### 4.3. Reliability of Delivery

**Figure 16.**
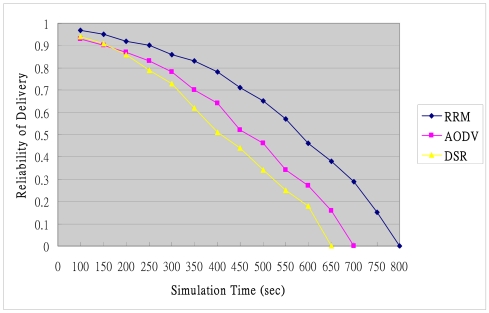
Average reliability of delivery.

The reliability of delivery falls with the increase in operating time due to the energy commitments to data processing, remaining sensor energy and hop count. The reliability falls more slowly in the proposed RRM mechanism than in the other tested mechanisms, as revealed in Figure 16, indicating that the routing path is more reliable, and that the energy consumption is more stable, in RRM than in the other mechanisms. Conversely, DSR and AODV consume more energy and incur greater packet loss than RRM. The flooding of routing overhead rises with the network scale, significantly affecting the performance of the entire network.

The proposed RRM mechanism includes intra-cluster routing and inter-cluster routing functions. The nodes periodically exchange routing information in intra-cluster routing, revealing the relationships among clusters from the local routing table in inter-cluster routing, which includes the next hop nodes and the edge of cluster. Accordingly the RRM mechanism has more routing paths available when compared to the DSR and AODV mechanism. The reliability of the proposed RRM mechanism is higher than that of the DSR and AODV mechanism.

The simulation results indicate that the average reliability of data delivery for RRM, AODV and DSR are 0.625, 0.498 and 0.434 respectively. The reliability of data delivery for RRM improved between 25% and 43%, which is higher than that of AODV and DSR mechanism. In addition, the reliability of the former is about 25% higher than that of the later.

### 4.4. Control Overhead

**Figure 17.**
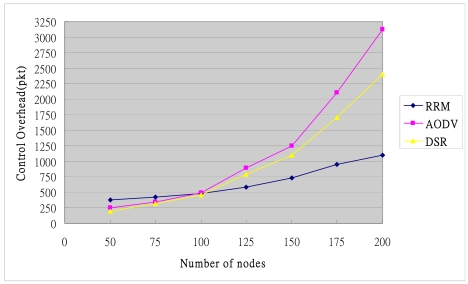
Control overhead.

The RRM inter-cluster routing is obtained by exchanging the relationships between clusters. Discovering inter-cluster routes can reduce the overhead of constructing and maintaining Inter-cluster routing. Conversely, the on-demand routing protocols, *i.e.*, AODV and DSR, adopt flooding for route discovery, so provide no previous routes to guide the packets to the destination. These methods not only have higher route discovery latency than RRM, but also have a higher overhead, depending on the range of flooding. The flooding of routing overhead rises with the size of network. Figure 17 indicates that the slope of curve increases more slowly in the proposed RRM mechanism than in the other mechanisms, revealing that RRM has the most stable control overhead, regardless of the number of sensor nodes. This is because RRM limits RREQ flooding within a cluster.

### 4.5. Average Latency

**Figure 18.**
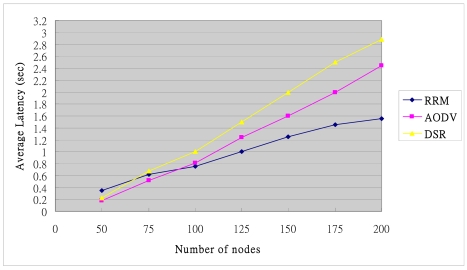
Average latency.

The DSR and AODV protocols only locate a route upon request when it is requested. The route discovery may delay the transmission of packets by queuing them in the buffer. Therefore, the latency exceeds that of RRM, which adopts a proactive scheme in clusters. In RRM, the packets can be delivered from one cluster to next cluster rapidly by intra-cluster routing information. Furthermore, each border node caches multi-path information. The RRM can rapidly change routing path when a path or sensor node fails. Figure 18 shows the average latency of the end-to-end communication. The source and destination pair, which measures the delay time of a packet between sent by the source and received by the destination, is selected randomly. The cluster track can be obtained on demand using unicast rather than broadcast, thus reducing the energy consumption.

### 4.6. Throughput

**Figure 19.**
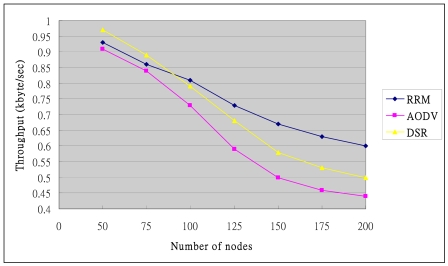
Throughput.

Figure 19 shows the measured throughput using three protocols, revealing that the throughput falls as the number of nodes in the network rises. Heavy routing overhead leads to the congestion and crowding out of the data packets. However, RRM exploits the cluster structure and multi-path feature to prevent bottlenecks. Therefore, the RRM slope decreases slowly. Hence, RRM still has a good throughput in a large network.

### 4.7. System Lifetime

**Figure 20.**
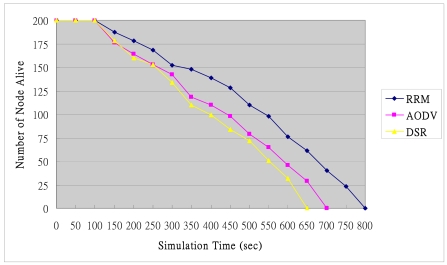
Number of node alive.

Figure 20 shows the number of live nodes. The cluster-structure design of RRM enables it to manage nodes effectively, minimize the average power consumption of a sensor node in a wireless sensor computing, and reduce unnecessary energy consumption.

## 5. Conclusions

The Reliable Routing Mechanism (RRM) combining the merits of cluster structure and reliability essential is proposed in this study. By constructing the cluster structure, the entire network can be divided into many clusters and the border nodes are then selected to cache the multi-path reliability information. This approach prolongs the average network lifetime. This approach can reduce the packets flooding throughout the entire network, and thus improve the routing reliability and efficiency. Simulation results indicate that the proposed RRM mechanism is an efficient multi-path routing methodology offering improved reliability, load balance and fault tolerance in large-scale sensor computing. The reliability of the proposed RRM mechanism is about 25% higher than that of the DSR and AODV mechanism.

## Figures and Tables

**Table 1.** N33's routing information.

**Cluster ID**	**Dest. ID**	**Next**	**Metric**	**Seq. No**	**Reliability**
C2	N_24_	N_34_	2	N_24_-200	0.94
C1	N_15_	N_31_	2	N_15_-100	0.96
C1	N_16_	N_31_	2	N_16_-110	0.95
C4	N_44_	N_35_	3	N_44_-200	0.93
C4	N_44_	N_36_	4	N_44_-320	0.90
C3	N_31_	N_31_	1	N_33_-110	0.97
…	…	…	…	…	

## References

[1] Huang Y.M., Hsieh M.Y., Chao H.C., Hung S.H., Park J.H. (2009). Pervasive, Secure Access to a Hierarchical-based Healthcare Monitoring Architecture in Wireless Heterogeneous Sensor Networks. IEEE J. Select. Areas Commun..

[2] Hsieh M.Y., Huang Y.M., Chao H.C. (2007). Adaptive Security Design with Malicious Node Detection in Cluster-Based Sensor Networks. Comput. Commun..

[3] AboElFotoh H., Iyengar S., Chakrabarty K. (2005). Computing Reliability and Message Delay for Cooperative Wireless Distributed Sensor Networks Subject to Random Failures. IEEE Trans. Reliab..

[4] Jiang Z., Wu J., Yang D. (2008). A New Fault Information Model for Fault-Tolerant Adaptive and Minimal Routing in 3-D Meshes. IEEE Trans. Reliab..

[5] Su B.L., Wang M.S., Huang Y.M. (2008). Localized and Load-Balanced Clustering for Energy Saving in Wireless Sensor Networks. Int. J. Commun. Syst..

[6] Zarei M. Reverse AODV Routing Protocol Extension using Learning Automata in *Ad hoc* Networks.

[7] Bai R., Singhai M. (2006). DOA: DSR over AODV Routing for Mobile *Ad hoc* Networks. IEEE Trans. Mobile Comput..

[8] Chen J.L., Lu H.F., Lee C.A. (2006). Autonomic Self-Organization Architecture for Wireless Sensor Communications. Int. J. Netw. Manag..

[9] Hoffmann G.A., Trivedi K.S., Malek M. A Best Practice Guide to Resource Forecasting for Computing Systems. IEEE Trans. Reliab..

[10] Sharma G., Mazumdar R.R. (2006). A Case for Hybrid Sensor Networks. IEEE/ACM Trans. Netw..

[11] Zhang Z.H., Ma M., Yang Y.Y. (2008). Energy-Efficient Multihop Polling in Clusters of Two-Layered Heterogeneous Sensor Networks. IEEE Trans. Comput..

[12] Bonivento A., Fischione C., Necchi L., Pianegiani F., Sangiovanni-Vincentelli A. (2007). System Level Design for Clustered Wireless Sensor Networks. IEEE Trans. Ind. Inform..

